# Quality of work life and factors associated with it among nurses in public hospitals, Iran

**DOI:** 10.1186/s42506-019-0029-2

**Published:** 2019-11-29

**Authors:** Pouran Raeissi, Mohammad Reza Rajabi, Elahe Ahmadizadeh, Kourosh Rajabkhah, Edris Kakemam

**Affiliations:** 1 Department of Health Services Management, School of Health Management and Information Sciences, Iran University of Medical Sciences, Tehran, Iran; 2 Department of Nephrology and Cardiology, Faculty of Medicine, Shahed University, Tehran, Iran; 3 Department of Health Management and Economics, School of Public Health, Tehran University of Medical Sciences, Tehran, Iran; 4 Health Services Management Research Center, Institute for Futures Studies in Health, Kerman University of Medical Sciences, Kerman, Iran; 5 Iranian Center of Excellence in Health Management, School of Management and Medical Informatics, Tabriz University of Medical Sciences, Tabriz, Iran

**Keywords:** Quality of work life, Iran, Nurses, Public hospitals

## Abstract

**Background:**

There is an acute shortage of nurses worldwide including Iran. Quality of work life is important for nurses as it affects the safety and quality of care provided for patients as well as organizational factors. The aim of this study was to describe the status of quality of work life and to explore its predictors among nurses in Iran.

**Methods:**

A cross-sectional study was conducted on 2391 nurses in 85 Iranian public hospitals, selected through the convenience sampling. Data were collected using demographic information and the quality of work life questionnaires.

**Results:**

The mean score for total quality of work life was 2.58, indicating a low level of self-reported quality of work life, with 69.3% of nurses dissatisfied with their work life. The major influencing factors were inadequate and unfair payment, lack of solving staff problems by organization and poor management support, job insecurity, high job stress, unfair promotion policies, and inadequate involvement in the decision-making. Significant predictors in the multivariate analysis for lower quality of work life were male gender, being single, older age, having lower educational levels, and working in teaching hospitals.

**Conclusion:**

The quality of nursing work life was at a low level and needs improvement interventions. The predictors identified allow for more targeted interventions. Nursing managers and policymakers should develop and implement successful strategies appropriately to improve the quality of work life. This includes the payments, organizational and managerial support, job security, fair promotion policies, and measures to reduce job stress.

## Introduction

Quality of work life (QWL) is a multifaceted variable which shows a worker’s feeling about various dimensions in regards to his/her job. These involve the job content, workplace conditions, enough and fair recompense, job promotional opportunities, duty discretion, involvement in decision-making processes, job safety, occupational stress, organizational security in employment and individual relations, and work life stability [1–3].

From a nursing point of view, Brooks defined the QWL as “the degree to which registered nurses are able to satisfy important personal needs through their experiences in their work organization while achieving the organization’s goals”. Hence, the idea of staff satisfaction is a broad concept which includes issues that are more crucial than merely giving some jobs and wages to individuals. Rather, it involves endowing people with some accommodations where they feel relaxed, wanted, and comfortable [4].

Some studies have revealed that QWL affects the performance and conscientiousness of workers in different sectors, involving healthcare settings [5, 6]. A high QWL is necessary to attract new staff and retain a workforce [2]. Therefore, health organizations are looking for methods to deal with issues of employment and retention by attaining a high level of QWL [1]. Positive results of QWL include improving organizational commitment and job satisfaction, increasing the quality of care, improving the productivity of individuals as well as the organization, and decreasing burnout and individual and organizational turnover [3, 6–8].

QWL among nurses in different countries varies from low level to moderate level. Akter et al. (2018) reported that the QWL as perceived by nurses in Bangladesh was at moderate level [9]. Findings from a study conducted in Saudi Arabia indicated that 52.4% of nurses, particularly primary health care nurses, were dissatisfied with their QWL [1]. Recent studies in Iran showed that between 70.8 and 81·2% of nurses reported that their QWL was low [7, 10]. A recent study in Ethiopia showed that 67.2% of the nurses were dissatisfied with the quality of their work life [11].

Studies on QWL indicated various factors that influence the QWL of nurses. One of these factors was the imbalance between work and life [1]. The major sources of low QWL identified were hectic work schedules, poor staffing, lack of autonomy in decisions, doing the tasks that are not related to nursing, lack of professional development opportunities, inappropriate working environment, and inadequate salary [1, 3, 11, 12]. Apart from these issues, management practices, relationship with colleagues, professional development chances, and the work conditions are other factors that affect the QWL of nurses in the work context [1–3, 7]. Studies have shown the effect of occupational development chances such as the promotion criteria and pursuing education on the QWL by nurses [1, 3, 5]. Regarding the work environment, the findings of prior studies indicate that nurses were not content with the safety in the hospitals [3, 13]. Payment and the status of nursing were other key factors in the literature influencing the QWL of nurses [1, 9].

In Iran, most of the employees in hospitals are nurses. According to a report issued by the Iranian Ministry of Health (2018), 140,000 nurses are working in Iranian hospitals. However, approximately 260,000 nurses are needed in order to maintain ideal levels of health care [14]. The most important reasons for the scarcity of nursing staff in Iran’s health care system involve poor nursing work conditions, lack of resources, inappropriate workload, unequal nurse-patient ratio, high bureaucracy, poor management support, and low salary [14, 15]. As the services in the hospitals are increased, this shortage of nurses also becomes more noticeable [16]. Hence, an understanding of QWL and related factors among nurses can inform improvement strategies. Additionally, the findings of this study may aid hospital administrators to address QWL among nurses and design and implement resilience strategies. The aim of the present research was to describe the state of QWL and explore factors associated with it among nurses in Iran.

## Methods

### Study design

We used a cross-sectional survey design.

### Setting

This study included 9 centers randomly selected from 31 Iranian provincial centers (or capitals). These provinces are as follows: Tehran, Ardabil, Kurdistan, Karaj, Kerman, Lorestan, Ilam, Urmia, and Tabriz. The selected provincial centers had a total of 110 public teaching and non-teaching hospitals. All hospitals with more than 100 beds were eligible for inclusion. Hence, 97 hospitals were approached for inclusion in the study, with 85 hospitals willing to participate.

### Study sample

A convenience, non-probability sample of all nurses working at hospitals was invited to participate in the study. Based on the results of a previous study among hospital nurses, the mean score of QWL was chosen as the primary outcome parameter and used to estimate the required sample size (mean = 2.53; SD = 0.52) [3], with confidence limits and precision set at 5% and 0.02, respectively. This resulted in sample size estimates of 2600 participants. We expected 70% response rate, so, 30% were added to compensate for dropouts. Thus, a total of 3380 were distributed among nurses.

### Inclusion and exclusion criteria

All full-time clinical nurses on a rotating roster over the previous month in these identified hospitals were approached for inclusion. Nurses who were new to the organization (less than 1 year of employment) were excluded to introduce a wash-out period to ensure responses related to the current organization.

### Data collection

The data were collected between Jun 2017 and July 2018. A total of 3380 printed anonymous questionnaires were handed personally (by authors) to nurses, and 2501 questionnaires were returned, with a response rate of 74.0% (58–87%) across hospitals. Review of the questionnaires indicated that 110 (4.4%) were incomplete and were therefore excluded. A total of 2391 questionnaires were assessed providing a response rate of 71.7%.

### Study tool

The survey tool used in the study included demographic information and questions relating to QWL. The following demographic variables were assessed: age, gender, marital status, clinical experience (years), work hours (per week), wards, income (per month), educational level, and type of employment (full-time permanent, full-time contract, bonded).

A QWL questionnaire was designed by Mosadeghrad to measure QWL [3]. It includes 36 items covering nine dimensions of participation and involvement, job promotion, solving staff problems by organization, communication, motivation for work, job security, wages and salaries, job stress, and job pride (four items in each domain) that collectively form the total QWL. Items were measured using a 5-point scale: ranging from 1 (very low) to 5 (very high), with higher scores indicating higher QWL. All dimensions demonstrated satisfactory internal consistency. Reliability in the present study was estimated with Cronbach α (0.71–0.82). The mean score across questions for each domain and the total perception of QWL are reported. All items related to job stress were reversely scored prior to further analysis so that lower scores reflected higher status of QWL. The range of total score was between 36 and 180, which in order to normalize the range on 1–5 scales for total score of QWL, the sum of raw scores of the total QWL was divided by the number of items. Scores between 2 and 2.75 on the total scale show low, scores between 2.76 and 3.50 show moderate, and scores between 3.51 and 5 indicate high [12].

### Data analysis

Data analysis was carried out using IBM SPSS version 20 (SPSS, Inc., Chicago, IL). Descriptive statistics were used to present the demographic data of the nurses and total QWL and its subscales. Multiple linear regression analysis was applied to identify the factors significantly associated with QWL. *P* value less than or equal to 0.05 was considered significant.

## Results

### Sample demographics

Demographic information is presented in Table 1. The majority of nurses were females (70.4%) and married (63.1%). About 43% of the sample were between 21 and 29 years (42.9%), with mean age of 31.26 (SD = 7) years. More than 40% of nurses worked in general wards. Respondents’ mean of work experience were 7.97 ± 6.5 years. About 60% had work experience ≥ 5 years (59.9%) and educated to a bachelor degree level (57.2%). 47.7% of nurses had permanent employment with more than half working overtime routinely (60.0%). Approximately, 80% of the participants worked in teaching hospitals and 67.2% of them received a monthly income between 15,000,000 and 30,000,000 Rials (US $1 = 45,000 Rials).

**Table 1:** Characteristics of nurse participants, Iran Public Hospitals, 2017–2018 (*n* = 2391)

Characteristic	*n* (%)
Gender	
Male	708 (29.6)
Female	1683 (70.4)
Marital status	
Single	887 (37.1(
Married	1504 (62.9)
Age (years)	
21–29	1026 (42.9)
30–39	957 (40.0)
≥ 40	408 (17.1)
Work experience (years)	
< 5	960 (40.2)
5–10	712 (29.8)
> 10	719 (30.1)
Level of education	
Associate degree/diploma	88 (3.7)
Bachelor degree	1367 (57.2)
Master degree or higher	936 (39.1)
Employment status	
Permanent	1141 (47.7)
Contract	722 (30.2)
Bonded	528 (22.1)
Work hours (weekly)	
Normal (≤ 44 h)	956 (40.0)
Overtime (> 44 h)	1435 (60.0)
Ward	
Critical care units	534 (22.3)
Emergency ward	454 (19.0)
General wards	1017 (42.5)
Others wards	386 (16.2)
Income per month (Rials)	
< 15.000.000	204 (8.5)
15,000,000–3,000,000	1606 (67.2)
> 3,000,000	581 (24.3)
Teaching status of hospital	
Teaching	1903 (79.6)
Non-teaching	488 (20.4)

### State of QWL

The mean and the SD of the scores of the nine dimensions and level of each dimension are reported in Table 2. The mean score of nurses QWL was 2.58 on a scale of 5 implying that total level of QWL was low. The total scores ranged from 1.44 to 4.06 (possible range 1–5). Similarly, mean scores on all domains ranged from 2.21 to 3.11 with scores of “motivation for work” (3.11 ± 0.69) the highest reported and “wages and salaries” (2.21 ± 0.51) the lowest.

**Table 2:** Mean scores and level of Quality of work life and its dimensions

Scales	Mean (SD)	Low QWL	Moderate QWL	High QWL
Participation and involvement	2.78 (0.57)	55.9	37.5	6.6
Job promotion	2.75 (0.50)	62.7	35.1	2.2
Solving staff problems by organization	2.28 (0.59)	83.5	13.0	3.5
Communication	2.60 (0.85)	62.8	29.4	7.8
Motivation for work	3.11 (0.69)	34.5	53.2	12.3
Job security	2.41 (0.85)	73.3	19.8	6.9
Wages and salaries	2.21 (0.51)	91.8	6.0	2.2
Job proud	2.58 (0.49)	69.1	26.7	4.2
Job stress	2.49 (0.57)	75.2	22.8	2.0
Total WQL	2.58 (0.40)	69.3	29.6	1.1

*SD* standard deviation, *QWL* quality of work life

The majority of participating nurses indicated they were not satisfied with items in the dimension of wages and salaries. Over 54% reported that their payment was not enough given the job market condition and the nature of roles they perform. More than three quarters of nurses believed that their wages and salaries within the organization (78.8%) and among similar organizations (77.5%) were not fair and there was inequality in payments.

In terms of job stress, around three fourths (73.5%) of nurses believed that their jobs were not safe and had negative effects on their physical and mental health. In connection with the dimension of solving staff problems by organization, over 60% of the participants reported that hospitals do not pay attention to the problems of nurses and do not try to meet their personal needs. Regarding the job promotion, more than half of the respondents (52.2%) believed that they had no opportunity to be involved in decisions making. In terms of professional development opportunities, 57.6% claimed that their organizations do not provide sufficient chance for career advancement. Regarding the communication, approximately 50% of nurses stated that they have poor communication with their managers and co-workers such as doctors and nurses. In the dimension of job security, 63.4% of participants believed that organizational rules did not provide job security for nurses and they did not receive sufficient supervision from their manager/supervisor.

Overall, 69.3% of nurses reported that their QWL was low. The majority of nurses were dissatisfied with their wages and salaries (91.8%), solving staff problems by organization (83.5%), job stress (75.2%), and job security (73.3%).

### Predictors of WQL

The multiple linear regression analysis of predictor variables for QWL is shown in Table 3. Older nurses had significantly higher mean of QWL than younger nurses (*B* = 0.013, 95% CI − 0.006 to 0.017, *P* = 0.038). Male nurses had lower QWL than female nurses (*B* = − 0.045, 95% CI − 0.080 to − 0.009, *P* = 014) and those who were married had higher QWL than single nurses (*B* = − 0.035, 95% CI − 0.071 to − 0.001, *P* = 0.008). Nurses with a master’s degree and PhD degree experienced higher QWL than those with a bachelor’s degree (*B* = − 0.054, 95% CI − 0.087 to − 0.021, *P* = 0.001). However, no significant association was observed for associate degree/diploma. Nurses working in teaching hospitals had significantly higher stress than those in non-teaching hospitals (*B* = − 0.085, 95% CI − 0.125 to − 0.045, *P* < 0.001).

**Table 3:** Multiple regression analysis of factors associated with quality of work life (*n* = 2391)

Factors	*B*	SE	95% CI	*p* value
Constant	2.19	0.11	[1.97 to 2.41]	< 0.001
Age	0.013	0.0029	[0.006 to 0.017]	0.038*
Clinical experience	− 0.005	0.0030	[− 0.011 to 0.001]	0.085
Work hours	− 0.001	0.0007	[− 0.002 to 0.001]	0.232
Gender (reference: female)				
Male	− 0.045	0.0181	[− 0.080 to − 0.009]	0.014*
Marital status (reference: married)				
Single	− 0.035	0.0185	[− 0.071 to 0.001]	0.008*
Educational status (reference: Master and PhD degree)				
Associate degree/diploma	− 0.059	0.0446	[− 0.146 to 0.029]	0.189
Bachelor degree	− 0.054	0.0169	[− 0.087 to − 0.021]	0.001*
Employment status (reference: bonded)				
Permanent	− 0.022	0.0268	[− 0.075 to 0.031]	0.411
Contract	− 0.034	0.0240	[− 0.081 to 0.014]	0.162
Income (reference: > 30,000,000)				
< 15,000,000	− 0.051	0.0335	[− 0.117 to 0.015]	0.129
15,000,000–30,000,000	− 0.017	0.0200	[− 0.056 to 0.022]	0.401
Ward (reference: other wards)				
Critical care units	− 0.025	0.0264	[− 0.077 to 0.027]	0.345
Emergency wards	− 0.045	0.0273	[− 0.098 to 0.009]	0.101
General wards	− 0.051	0.0245	[− 0.107 to − 0.014]	0.090
Teaching status (reference: non-teaching)				
Teaching	− 0.085	0.0203	[−0.125 to − 0.045]	< 0.001*
*R* = 0.153, *R*^2^ = 0.023, adjusted *R*^2^ = 0.019, *F* = 5.72				

*CI* 95% confidence intervals

*Significant

## Discussion

This study demonstrated that the mean score of QWL was 2.62, which was a low level. Likewise, 69.3% of nurses perceived their QWL as low. These results are in line with other similar researches in Iran [3, 17], while the QWL as perceived by nurses in Bangladesh, China, Canada, and Taiwan was at moderate level [2, 9, 18, 19]. Also, the results of this study were consistent with the results of three studies in Ethiopia, Egypt, and Nigeria [11, 20, 21]. There may be differences related to societal values, economic conditions, employment structures, and management of Iranian hospitals compared to other countries.

The low QWL in this study may be due to a reform made in the Iranian health system with the current implementation of the Health Sector Evolution Plan (HSEP). Inequity in wages and workload are the consequences associated with this reform [22]. Salarvand et al. came to the conclusion that the HSEP has led to increasing workloads, capped salaries, staff shortages, and negative impacts on physical, mental, social, and professional outcomes which were previously identified as factors that decreased hospital nurses’ work satisfaction. Furthermore, the implementation of this reform has intensified the burnout among nurses [23].

The most important reasons of low QWL were inadequate and unfair payment, lack of solving staff problems by organization and absence of management support, job insecurity, high job stress, unfair promotion policies, and lack of participation in decision-making. These results are consistent with Mosadeghrad’s studies (2013, 2011) in Iran [3, 17].

Salaries and financial supports were identified to be determining factors in the dissatisfaction of respondents with their QWL. Though it has been demonstrated that salary is not the main incentive for employees [24], behavioral theorists such as Herzberg and Maslow show that fulfilling basic needs is necessary because people cannot focus on their higher needs unless basic their needs are satisfied [1, 5]. Accordingly, some studies have shown that wages, financial benefits, and equality in pay were very crucial to nurses [1, 5, 25], and the lack of such benefits may have effect on the job satisfaction, performance, and commitment of nurses [26, 27].

The nurses in this study were not satisfied with the chances for professional development (i.e., opportunity to further education, career advancement, and access to continuing education). This result is consistent with previous nursing studies in Iran [3, 28]. Hart’s study showed that respondents who participated in an educational course were less likely to quit their positions than those who did not attend in any program [29]. Another factor that was reported in prior studies was nurses’ dissatisfaction with career advancement [1, 28]. Similarly, Rout’s study in the north-west of England revealed insufficiency of opportunities for career development and low amounts of job satisfaction among the nurses [30]. A study in Ethiopia reported that the promotion opportunities and professional growth had an influential impact on the QWL of nurses [11].

Lack of solving staff problems by the organization and the paucity of managerial support were found to be problematic issues in the QWL. These problematic areas included weakness of supervision, feedback, involvement in making decisions, and respect shown by management. Sufficient procedural guidelines and working policies are also influential in this regard. Approximately, 78% of nurses believed that their hospital managers do not reply to staffs’ concerns in Atefi et al.’s study in Iran [28]. Similarly, Mirzabeigi et al. concluded that Iranian nurses complained about managerial issues [31]. In former researches, nursing administration practices were identified to be related to the quality of care, personnel productivity, staff satisfaction, and the intention to quit or stay [32–35].

Another key factor in the dissatisfaction of nurses with their QWL was high job stress. Several researches concluded that stress in the work environment decreases the level of QWL of nurses [3, 9, 17]. A study recently (2019) reported long working hours, shift work, under-staffing, inadequate pay, discrimination at work, unsupportive management, and poor communication as the major sources of occupational stress among Iranian nurses [36]. This type of stress may have deleterious impacts on a nurse’s physical as well as mental and emotional health [37]. Workplace stress can have negative impact on the quality of care [38].

Job insecurity was another main factor in the dissatisfaction of the QWL of nurses. This issue was identified in Mosadeghrad et al.’s study [17] as a key factor negatively influencing the QWL of hospital staff. Hsu and Kernohan also identified job insecurity as a factor affecting the QWL of nurses [5].

Our findings revealed that being younger, single, and male, having a lower educational level, and working in a teaching hospital were all associated with lower QWL levels. In line with other studies, male respondents had significantly lower mean scores of QWL than female nurses [3, 12]. The generally higher scores may be explained by the fact that males are the partner who is responsible for earning money to support the family. Another reason could be that in Iran, nursing has been viewed as a profession for women and it is traditionally more acceptable for women than for men [28].

Older nurses had significantly higher mean scores of QWL than younger nurses. Many studies have found that older respondents are more satisfied than younger [3, 9, 12, 28]. This may be related to the capability of older nurses in their adaptation to the workplace [39]. Apart from that, older staff are appreciated and understood by managers, and as a result, they appear to be more satisfied.

With regards to the marital status, single nurses had significantly lower mean scores of QWL than the others, a finding which is consistent [1, 3, 12] with former researches. This result may be attributable to the lack of the skills of the single nurses in coping with the challenges in the workplace and possibly to the fact that most of the married nurses lived with their families, which in turn increased their job satisfaction significantly [28].

This study found that the QWL of nurses with higher educational status was higher than nurses with lower educational status. These findings are in line with the results reported by studies conducted in Bangladesh and Ethiopia [9, 11]. However, this result is not parallel with the results of previous nursing studies in Iran [3, 12]. It is likely that nurses with higher education levels have higher expectations of their working life and consequently experience more emotional exhaustion when their work environment does not meet their expectations.

Working in teaching hospitals was also identified as a predictor of low QWL, as has been reported in a previous study [26]. Longer working hours contribute to greater burnout [40] and higher workloads in teaching hospitals, because of the requirements of student teaching programs and workplace demands [26].

### Limitations

There are some limitations of the present study. First, the design of the present study was cross-sectional; future researches should apply other designs to confirm the results and explore the causality of relationships. Second, our study was conducted among nurses working in public hospitals, and therefore, the findings might not be generalized to private hospitals. Finally, in the present study, QWl was measured by self-report only which may not reflect the true picture of the QWL.

## Conclusions and recommendations

The majority of the nurses were dissatisfied with their QWL. The most important reasons for the low QWL of the nurses were inadequate and unfair payment, lack of solving staff problems by the organization and poor management support, job insecurity, high job stress, unfair promotion policies, and inadequate involvement in the decision-making. Our results have implications for interventions targeted at improving nurse QWL in Iranian public hospitals by providing evidence-based practice guidelines for nursing managers. These results could inform evidence-based policies and practices through interventions aimed at improving QWL and increasing resilience. QWL of nurses could be increased by international benchmarking techniques by managers, including more support from nurses, fair promotion policies, the participation of nurses in decision-making, reduction of stress, consideration of payment, and boosting the job security of nurses.

## Data Availability

The datasets used and/or analyzed during the current study are available from the corresponding author on reasonable request.

## Abbreviations

HSEP: Health Sector Evolution Plan
QWL: Quality of work life
